# Evolution of the species Ovis aries

**DOI:** 10.18699/vjgb-26-70

**Published:** 2026-07

**Authors:** T.N. Khamiruev

**Affiliations:** Research Institute of Veterinary Medicine of Eastern Siberia – Branch of the Siberian Federal Scientific Centre of AgroBioTechnologies of the Russian Academy of Sciences, Chita, Russia

**Keywords:** evolution, genetics, mouflon, domestication, domestic sheep, migration, haplogroup, эволюция, генетика, муфлон, доместикация, домашняя овца, миграция, гаплогруппа

## Abstract

The sheep was one of the first domesticated animals in Neolithic Southwest Eurasia. The presented study suggests that the domestication of sheep occurred on the Anatolian plateau, to the northwest of the commonly accepted boundaries of the Fertile Crescent, rather than within it. However, many aspects of the domestication process (the specific place, time, and history after domestication) have remained not fully understood. The review examines in detail the complex origin, traces the primary role of the Asian mouflon (Ovis gmelini) as an ancestor, and also discusses controversial aspects of the contribution of other wild Ovis species. It reveals the history of the introduction and migration of sheep in the world, and summarizes the current scientific understanding of the phylogenetic relationships between populations of wild mouflons and domestic sheep (Ovis aries). A multifaceted process of domestication is considered, and the proposed evolutionary mechanisms are discussed, such as the domestication syndrome and hypotheses about thyroid hormones, as well as the human-mediated selection of key phenotypic traits. The article analyzes the results obtained using various genetic markers, including mitochondrial DNA haplogroups. Phylogenetic analysis using mitochondrial DNA has been successfully applied to identify the phylogeographic patterns and divergence times, from the early Neolithic to the Middle Ages, of sheep migration from the domestication center to Asia, Europe, and Africa. Domesticated sheep, having survived and endured extreme climate changes that occurred in the last post-glacial period, became the ancestors of modern local sheep breeds. Starting from the seventh millennium BC, domesticated sheep were brought to the Caucasus, Central Asia, and Europe. The spread of sheep in Asia began from the Middle East to the Mongolian Plateau and the Indian subcontinent, then to the north and southwest of China. In Russia, the territory of which covers a significant part of Eurasia, a unique breed diversity of sheep has been developed, with haplogroup B typical for breeds of the European-type origin (western geographic regions), and haplogroup A of the Asian-type sheep (eastern geographic regions).

## Abstract (ru)

Овца – одно из первых одомашненных животных в неолитической Юго-Западной Евразии. Представленное исследование позволяет предположить, что одомашнивание овец произошло на Анатолийском плато, к северо-западу от общепринятых границ Плодородного полумесяца, а не внутри них. Однако многие аспекты процесса доместикации (конкретное место, время и история после доместикации) остаются до конца невыясненными. В обзоре подробно рассматривается филогенез, прослеживается основная роль азиатского муфлона (Ovis gmelini) как предка, а также обсуждаются спорные аспекты вклада других диких видов Ovis, раскрывается история интродукции и миграции овец в мире, обобщается современное научное понимание филогенетических связей между популяциями диких муфлонов и домашних овец (Ovis aries). Рассматриваются многогранный процесс приручения, обсуждаются предлагаемые эволюционные механизмы, такие как синдром одомашнивания, и гипотезы о гормонах щитовидной железы, а также опосредованный человеком отбор ключевых фенотипических признаков. В статье анализируются результаты, полученные с помощью различных генетических маркеров, включая гаплогруппы митохондриальной ДНК. Филогенетический анализ с использованием митохондриальной ДНК был успешно применен для выявления филогеографических закономерностей и времени дивергенции с раннего неолита и до средневековья, миграции овец из центра одомашнивания в Азию, Европу и Африку. Одомашненные овцы, пережившие и выдержавшие экстремальные изменения климата, происходившие в последний послеледниковый период, стали родоначальниками современных местных пород овец. Начиная с седьмого тысячелетия до нашей эры одомашненные овцы были завезены на Кавказ, в Центральную Азию и Европу Распространение овец в Азии началось с Ближнего Востока на Монгольское плато и Индийский субконтинент, затем на север и юго-запад Китая. В России, территория которой занимает значительную часть Евразии, выведено уникальное породное разнообразие овец, при этом гаплогруппа B типична для пород овец европейского типа происхождения (западные географические регионы), а гаплогруппа A – для овец азиатского типа (восточные географические регионы).

## Introduction

The genus Ovis has existed for about 8.31 million years and includes eight living species: domestic sheep (O. aries), argali (O. ammon), Asiatic mouflon (O. orientalis), European mouflon (O. musimon), urial (O. vignei), bighorn sheep (O. canadensis), thinhorn sheep (O. dalli) and snow sheep (O. nivicola) (Rezaei et al., 2010). At the same time, the origin and “wild” status of the European mouflon are the subject of significant discussions (Hiendleder et al., 2002; Chessa et al., 2009). P. Mereu and colleagues (2025) believe that it should be classified as a subspecies of the Asian mouflon. It has been historically believed that European mouflons, especially those living in Corsica and Sardinia, are remnants of an ancient population of European wild sheep (Poplin, 1979). However, archaeological evidence indicates that they were brought to Cyprus by humans in the early Neolithic (about 10,000 years ago), and then to Corsica and Sardinia (Vigne et al., 2011). These populations were far from domesticated in the modern sense, and were kept in fenced areas only for protection from predators and to provide a constant, easily accessible source of meat and hides with minimal interaction between humans and animals (Barbato et al., 2022; Portanier et al., 2022; Mereu et al., 2024).

The domestic sheep O. aries Linnaeus is among the first domesticated species of livestock. Domestication occurred about 12,000 years ago in the Middle East, in the Zagros Mountains region (Her et al., 2022; Mereu et al., 2024), at the same time, many aspects of the domestication process, such as the exact place and time, remain unclear (Kaptan et al., 2024). Subsequently, as a result of human migration, sheep spread throughout Eurasia and under the influence of natural and artificial selection formed various populations (Wang et al., 2025).

The origin of domestic sheep included the initial domestication and early divergence, followed by extensive post- Neolithic diversification. At the early stage of domestication, human involvement was minimal and was mainly focused on protecting and providing food for the sheep, rather than on intensive breeding aimed at developing specific traits (Portanier et al., 2022; Mereu et al., 2025). It is extremely important to understand this primitive phase of sheep domestication in order to distinguish the genetic changes that existed before domestication from those directly associated with selection carried out by humans. This historical context shows that domestication is not a single event, but a continuous process characte- rized by gradual accumulation of genetic, phenotypic, and physiological changes (Jackson et al., 2020; Mereu et al., 2024).

F. Teletchea and P. Fontaine (2014) proposed a classification with five levels of domestication: the first level corresponds to initial trials of acclimating wild animals in captivity. Then, when part of the life cycle is under control, the second level comes, where the most important thing is breeding in captivity. The third level occurs when the entire life cycle is under control, but the instincts of wild ancestors are still present. At the fourth level, domesticated animals differ significantly from their wild counterparts, and the fifth level leads to the creation of certain breeds. It is also worthy of note that different population groups within a single species can indeed exhibit different levels of domestication, even within the same geographic area (Teletchea, Fontaine, 2014; Zeder, 2015).

Despite intensive work using genomic data of modern sheep (Lv et al., 2022; Matyukov et al., 2023; Deniskova et al., 2025; Dossybayev et al., 2025), neither the exact location nor the wild ancestors involved in the first domestication process, nor the post-Neolithic demographic history of domestic sheep or mouflon populations have been clarified so far (Kaptan et al., 2024).

## Domestic sheep origin

One of the open questions is the origin of the gene pool of domestic sheep. Earlier studies pointed to several species of Asian sheep (O. ammon, Linnaeus, 1758; O. gmelini, Blyth, 1841; O. vignei, Blyth, 1841) as the probable ancestor of domestic sheep (Pedrosa et al., 2005; Sanna et al., 2015). However, recent genetic studies confirm the view that the Asian mouflon is the only ancestor of the domestic sheep (Chen et al., 2021); at the same time, according to D. Kaptan et al. (2024), this fact has not been fully established. More recent genomic studies indicate that the VPS13B gene shows signals of introgression from urials or mouflons, while argali contributed alleles to the lines of domestic sheep in Southeast Asia, introgressing the MSRB3 gene, which has been found to be specifically associated with variations in ear morphology (Cheng et al., 2023)

The O. gmelina species includes five subspecies: the Armenian mouflon (O. gmelini gmelini, Blyth, 1841), Isfahan mouflon (O. gmelini isphananica, Nasonov, 1910), Laristan mouflon (O. gmelini laristanica, Nasonov, 1910), Cyprus mouflon (O. gmelini ophion, Blyth, 1841) and Anatolian mouflon (O. gmelini anatolica, Valenciennes, 1856). Due to the complex intraspecific taxonomy of the Asian mouflon, the genetic contribution of various subspecies to the domestication of sheep remains unclear (Demirci et al., 2013; Li R. et al., 2021; Daly et al., 2025). According to D. Sanna et al. (2015), these subspecies represent local wild populations since the beginning of the Holocene, with the exception of the Cypriot mouflon, which was brought from mainland Anatolia around the 12th millennium BC.

S. Hiendleder et al. (2002) suggested that sheep were domesticated from two different subspecies in Turkey and western Iran (Middle East), corresponding to mtDNA lineages A and B of domestic sheep, respectively. Significant nucleotide diversity of mitochondrial lineages in this region confirms the geographical role in sheep domestication and indicates a more complex picture of domestication than previously assumed (Meadows et al., 2007; Machová et al., 2022).

Recent studies, including genetic and geographic data of Asian mouflons across their range, as well as modern and ancient sheep from Anatolia, Southwest Asia, Europe, and Africa, suggest that early sheep domestication probably originated in Anatolia and is associated with the Anatolian subspecies of the mouflon (O. gmelini anatolica) (Her et al., 2022; Atağ et al., 2024; Sandoval-Castellanos et al., 2024).

It is assumed that Anatolian and Cypriot mouflons in the past were subject to domestication according to the classification F. Teletchea and P. Fontaine (2014), corresponding to the first level (Sanna et al., 2015; Barbato et al., 2017). However, this is only a hypothesis for now, and the genetic connections between these mouflons, ancient and modern domestic sheep remain unclear.

Another largely unresolved question is the history of domestic sheep breeds. Modern sheep breeds are divided into two main geographical groups: Europe and Asia–Africa (Naval- Sanchez et al., 2018; Li X. et al., 2020). This eastern and western genetic division can be traced back to 7,000–6,000 BCE, indicating their early diversification. Such a division is also observed in modern groups of mitochondrial haplotypes of sheep: European sheep mainly carry haplotype B (>50 % of the world’s sheep population), while Asian sheep predominantly have haplotype A (34 % of the world’s sheep population) (Machová et al., 2022; Mereu et al., 2024).

Based on the analysis of polymorphism in the control region of sheep mtDNA, seven haplogroups were identified, two of which (F and G) have disappeared, while the other five are present in modern breeds (Wood, Phua, 1996; Chen et al., 2021). In addition to the mentioned haplogroups A and B, a third recognized phylogenetic branch, haplogroup C (9 % of the world’s sheep population), was discovered, which was found in local Portuguese sheep, as well as in individuals from the Caucasus, the Middle East, and Asia (Guo et al., 2005; Pedrosa et al., 2005; Pereira et al., 2006; Mereu et al., 2024). Then the fourth maternal lineage, haplogroup D, which is closest to O. gmelini anatolica, was identified (Demirci et al., 2013; Sanna et al., 2015).

Haplogroup E, identified through analysis of D-loop, CytB sequences, and the entire mitogenome, is the key mitochondrial lineage of the Iranian Asian mouflon (Mereu et al., 2025; Wang et al., 2025). The most recently discovered D and E haplogroups (<0.5 % of the world’s sheep population) are the rarest, having been identified only in animals from Turkey and the Caucasus (Tapio et al., 2006), Europe (Gáspárdy et al., 2021), Tibet (Liu et al., 2016), Iran (Rafia, Tarang, 2016). All haplogroups were presumably formed between 5 and 35 thousand years ago (Rezaei et al., 2010).

Scientific literature reports on the eighth “wild” mitochondrial lineage X, which is found in Cypriot, Anatolian, and Iranian mouflons and apparently is basal in relation to domestic haplogroups C and E (Guerrini et al., 2021; Mereu et al., 2025).

The results of ancient DNA studies have shown that Anatolian Neolithic sheep are more similar to modern European breeds, while the Kyrgyz Neolithic sheep are more similar to modern Asian breeds, suggesting an early formation of this division (Yurtman et al., 2021).

These patterns imply either the presence of several centers of domestication or a large heterogeneous precursor population that went through several independent “bottlenecks”. However, in the study by E. Yurtman et al. (2021), all modern breeds showed greater genetic similarity to each other than to Neolithic sheep, indicating significant breed mixing in the post-Neolithic period among continental sheep populations, including possible introgression from wild sheep into domestic flocks, as well as the spread and breeding of sheep with desired traits across the continents (Deng et al., 2020; Cheng et al., 2023).

Introgression from wild relatives into the domestic sheep population allowed the latter to adapt to various environmental conditions and spread throughout the world, undergoing genetic improvements in various production systems. General signals of allele introgression have been established in the genes related to olfaction (ADCY3 and TRPV1), and in the PADI gene family, including PADI2, which is associated with innate immunity.

Further analysis of whole-genome sequences showed that introgressed alleles in a specific region of PADI2 (chr2: 248302667–248306614) correlate with resistance to pneumonia. This allowed sheep to adapt to various climatic and environmental conditions after domestication (Ciani et al., 2020; Cao et al., 2021). As a result, many unique breeds have emerged, and the extensive variations observed in both local and improved breeds highlight the genomic diversity, adaptive traits, and important productive characteristics inherent in domestic sheep (Li X. et al., 2020; Cao et al., 2021; Alipanah et al., 2025). The differentiation of local sheep populations into breeds became more pronounced from the 18th century due to the use of systematic breeding with clearly defined goals (Ciani et al., 2020).

## Phenotypic changes in domestic sheep

In the transition from wild ancestors, the mouflons, to modern domestic sheep, a number of morphological, physiological, and behavioral traits were formed, which led to the current phenotypic differences between wild and domesticated species. These traits, including brain and tooth size, the size and shape of ears and tail, spotted coloration, hormonal changes, and the length of the reproductive season, characterize many species that have been domesticated in various ways and for different reasons. Moreover, some of these traits apparently appeared selecting sheep with fat tails for their increased adaptability to desert conditions and as a valuable source of fat (Ahbara et al., 2022; McManus et al., 2025). Genes such as bone morphogenetic protein 2 (BMP-2) and platelet-derived growth factor D (PDGF-D) are potential genetic markers for tail shape and size (Luo et al., 2023; Jin et al., 2026). The T-box T (TBXT) transcription factor gene is involved in regulating tail length in mammals, including sheep (Kalds et al., 2022b). CT/CT genotype of the TBXT gene: c. [333G>C; 334G>T] was found in fat-tailed sheep breeds and was absent in long- and short-tailed breeds (Han et al., 2019).

The emergence of these phenotypic changes is not accidental, but represents a direct adaptation to environmental conditions, highlighting the strong selective pressure exerted by humans in the process of domestication. Analysis of these phenotypic shifts in combination with the identification of the underlying genes provides valuable insights into the genetic architecture of domestication and how human needs and environmental factors have shaped the evolution of the species O. aries.

Later introgressions from wild ancestors to domestic sheep contain loci with potential domestication genes PAPPA2, NR6A1, SH3GL3, RFX3 and CAMK4, which are associated with morphological, immune, reproductive, or productive traits (wool, meat, milk), NEURL1 – with a nervous reaction, PRUNE2 – with neurogenesis, USH2A – with hearing, AGTPBP1, CRTAC1 and RPGRIP1L – with the sense of smell, PAG11 and PAG3 – with placental viability (Chen et al., 2021), FAT3 – with adaptation to dry environmental conditions (Yang J. et al., 2016), PCDH15 – with the immune response (Atlija et al., 2016), PDGFD – with the tail shape (Li X. et al., 2020).

## Migration of domesticated sheep

Starting from the 7th millennium BC, domestic sheep were brought from Southwest Asia to the northeast, to the Caucasus (Chataigner et al., 2014), to Central Asia (Taylor et al., 2021) and to Europe (Arbuckle, Atici, 2013; Lv et al., 2015).

According to F.-H. Lv et al. (2015) and K. Machová et al. (2022), the spread of sheep across continents from the domestication center occurred according to the Figure

**Fig. 1.**
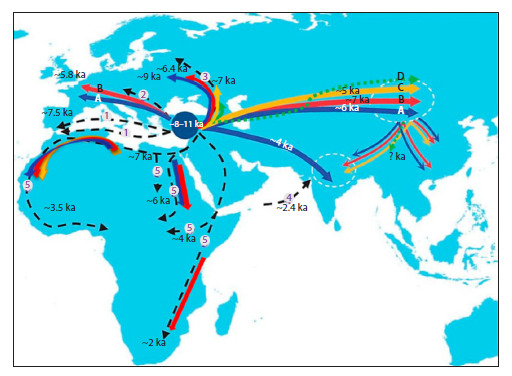
The main routes of sheep spread from the center of domestication (ka – thousand years BC) (Lv et al., 2015; Machová et al., 2022). A, B, C, D – paths of the main mitochondrial lineages (Lv et al., 2015; Liu et al., 2016). 1 – Mediterranean route (Ryder, 1984; Zeder, 2017); 2 – Danube route (Ryder, 1984; Zeder, 2017); 3 – route to Northern Europe (Tapio et al., 2006); 4 – routes of the ancient sea to the Indian subcontinent (Singh et al. 2013); 5 – routes to Africa (Gornas et al., 2011; Álvarez et al., 2013; Muigai, Hanotte, 2013; Resende et al., 2016).

There is a hypothesis about the existence of two routes for the spread of sheep from Anatolia to Europe: a continental route and a sea route through the Mediterranean Sea (Racimo et al., 2020; Brigand et al., 2022). The sheep of Northern France during the Neolithic era arrived in this territory along with the Linear Pottery culture, which, as is known, spread westward from the middle Danube to the Rhine and Seine basins, as well as northward and eastward to the Elbe and Vistula basins (Hachem, 2018; Auxiette, Hachem, 2021).

Iberian sheep of the Neolithic era were possibly brought by the Mediterranean Sea route (Kaptan et al., 2024). Later, the settlement of Northern Italy and Southern France took place. Then, along the Danube route, sheep spread to Central and Northern Europe (Larsson et al., 2024). Sheep appeared in the Alps just over 5,000 years ago, on the Iberian Peninsula (Spain, Portugal), 7,700–7,300 years ago (Chen et al., 2021). However, according to another hypothesis, there was a settlement of Northern Europe through the domestication center in the Caucasus (Tapio et al, 2006; Niemi et al., 2013).

Analysis of the spread of the lines in Eastern Eurasia showed the presence of two migratory waves of sheep that occurred 4.5–6.8 thousand years ago (lines A and B, about 6.4–6.8 thousand years ago; C, about 4.5 thousand years ago) (Lv et al. 2015).

According to the study by D. Cai et al. (2011), line A was the most numerous in Bronze Age Ancient China (95.5 %), and its population increased from west to east. It is assumed that sheep from the Near Eastern center of domestication migrated through the Caucasus and Central Asia, and settled in Northern and Southwestern China (lines A, B, and C), and then on the Indian subcontinent (lines B and C) (Lv et al., 2015).

The spread of sheep in Asia could have occurred through several routes, as it did in Europe (Singh et al., 2013). A combined phylogenetic analysis using ancient and modern sheep mitogenomes showed that Uzbekistan and the Mongolian Plateau (Altai) could have been the migration centers for the spread of sheep in East Asia. Through Uzbekistan and northwestern China, sheep spread to the middle and lower reaches of the Yellow River (around 4,000 BC), through the Mongolian Plateau (Altai) to the central part of Inner Mongolia (around 4,429–2,500 BC) (Yang L. et al., 2024). Earlier studies confirm that sheep spread to China from the Mongolian Plateau approximately 5,000–5,700 years ago. Then, about 2000–2600 years ago, sheep reached the Qinghai-Tibet and Yunnan-Guizhou Plateaus, following the migration routes of the Di-Qian people from the north to the southwest (Zhao X.Y. et al., 2017).

Sheep probably reached North Africa by two routes about 7,000 years ago. The first was a colonization settlement across the Mediterranean Basin, the second was via the Sinai Peninsula across the Red Sea (Zeder, 2017). There were several routes of settlement on the African continent: to the south to the Middle Nile valley, to the west to the central Sahara, and to the north to Libya. Another possibility is the spread of sheep from the Mediterranean along the northern coasts of Africa. In addition, direct trade connections between East Africa and the Arabian Peninsula are also suggested (Muigai, Hanotte, 2013). As in Europe, mitochondrial haplogroup B dominates in Africa, which is confirmed by the studies in different regions of the continent – South Africa (Horsburgh, Rhines, 2010), Sudan (Gornas et al., 2011), Kenya (Resende et al., 2016), West Africa and the Canary Islands (Álvarez et al., 2013).

The spread of sheep in Australia occurred in modern history in the 18th–19th centuries (Ciani et al., 2015). The first sheep brought by the Spanish to Central America were either finewool (West African fine-wool sheep) or coarse-wool (from the Iberian Peninsula), which were later crossed with Merinos and gave rise to the Creole type of sheep (Alonso et al., 2017).

Fine-wool sheep were brought to America from the Canary Islands by C. Columbus and the first colonizers and at the beginning of the seventeenth century along with slaves from other regions of West Africa, and their contribution to the gene pool of modern American fine-wool sheep is the most significant (Spangler et al., 2017).

The first sheep in Australia were brought from India, South Africa (fat-tailed) and Spain (Merinos) after 1788, as well as from the British Isles (Saxon Merinos, Southdowns and Romneys) after 1840 (Ryder, 1984). Thus, it can be assumed that the same haplotypes exist as in the populations from which the Australian breeds originated. This assumption was confirmed by a study conducted on 18 breeds kept in Australia, which revealed a 55 % prevalence of haplogroup B and a 45 % prevalence of haplogroup A (Meadows et al., 2005).

Based on the analysis of ancient samples, it has been established that modern breeds show greater genetic similarity to each other than to Neolithic sheep, which implies significant post- Neolithic mixing of sheep populations (Kaptan et al., 2024).

Originally, sheep breeding was mainly focused on meat production, while specialization in wool and milk arose in Asia (7–6 thousand years BC) and then in Europe (Becker et al., 2016; Deng et al., 2020). Specialization in wool production apparently originated in Southwest Asia and only then spread to Europe, as evidenced by research (Chessa et al., 2009) and the DNA analysis of European Bronze Age sheep (Sabatini et al., 2019). Archaeological findings also indicate the emergence of a new breed in Central Europe during the Late Stone Age. Comparison with earlier data confirmed an increase in the body size of sheep raised in the Bohemia and Moravia region (the territory of modern-day Czech Republic), as well as since the beginning of the Bronze Age in Hungary (Kyselý, 2016). Another example is the spread of Merino sheep from the Iberian Peninsula throughout Europe from the second half of the 15th century (Landi et al., 2019).

Early domestication highlights the fundamental importance of sheep in human history, as since then they have represented a crucial resource on a global scale, providing populations around the world with meat, wool, pelts, sheepskins, milk, and other essential materials (Kaptan et al., 2024; Mereu et al., 2024).

Early domestication was probably a less controlled process, based on the use of available wild populations rather than on intensive selection of a single homogeneous ancestral group. Therefore, the genetic diversity observed in modern sheep partially reflects ancient wild diversity, and is not exclusively the product of post-domestication mutation or selection. At the same time, the early radiation (about 810 thousand years ago) determined the variability of domestic sheep and mouflons (Mereu et al., 2025).

Thus, the process of sheep domestication should not be considered as a simplified model of domestication in the form of a single event from a homogeneous ancestral population, but as a more complex action involving the borrowing of diverse wild genetic backgrounds.

## Phylogenetic analysis of modern sheep breeds based on mtDNA

Subsequent breeding of domesticated sheep, along with natural and artificial selection, led to the emergence of at least 1,400 breeds, and the prolonged selective activity of humans played an important role in the development of the necessary productive and biological traits in sheep (Dymova et al., 2019). Comparative analysis of the genetic diversity of ancient and modern sheep contributes to a better understanding of the processes of the origin of these animals, domestication, and their migration, and also allows assessing the role of humans at different stages of the formation of modern biological diversity of sheep breeds (Lv et al., 2015).

In the study by X. Li et al. (2020), 36 local and 6 cultured breeds of sheep (O. aries) from Europe, Asia, Africa, and the Middle East were found to have a lower level of genomic diversity compared to their wild ancestors (O. orientalis). This indicates that a significant part of genomic variability was lost during and after domestication, while genomic diversity in local breeds of sheep has been preserved to a greater extent than in commercial breeds.

The formation of sheep breeds in Europe, according to E. Ciani et al. (2020), indicates that Greek sheep breeds acted as a barrier between Asian and European breeds. Apparently, Greece remained a “blind spot” on the evolutionary map of sheep migration to Western Europe, since the historical patterns of gene flow have not yet been assessed (Michailidou et al., 2025).

In Italian sheep breeds, haplogroup B predominated in the ancient samples (90 %), while haplogroup A was found in 10 % of them, which corresponds to the current distribution in modern sheep in Italy. This indicates that the current A/B ratio was formed back in the Middle Ages and is not the result of subsequent events, such as selective breeding (Gabbianelli et al., 2015). Eight of the most common local breeds of sheep in Bulgaria showed a high prevalence of European haplogroup B (95.2 %), while the remaining individuals were assigned to haplogroup A (4.8 %). The median-joining network showed that almost all haplotypes belonging to haplogroup B form a star-like network, indicating weak genetic differentiation and high gene flow among Bulgarian indigenous breeds (Kalaydzhiev et al., 2023).

The presence of haplogroups B and A was confirmed in two Hungarian breeds, Tsigai and Cikta, while haplogroup C was found only in the Cikta breed. At the same time, haplogroup B predominates, which is present at a level of more than 80 % in both breeds, confirming the presence of a common ancestral root (Gáspárdy et al., 2022). A homogeneous maternal origin of nine breeds of sheep from the Eastern Adriatic, characteristic of European breeds, was established in the studies of M. Ferencakovic et al. (2013); analysis of mitochondrial DNA revealed a high frequency of type B haplotypes in them.

Among the 27 analyzed Indian sheep breeds, three mtDNA lineages were identified, namely A, B, and C. Lineage A predominated among Indian sheep, whereas lineages B and C were observed at a low frequency. At the same time, the Mandya and Sonadi breeds differed significantly from other Indian breeds by a high proportion of line B, which presumably reached the Indian subcontinent not from the Mongolian Plateau, but via the Arabian Sea route (Kamalakkannan et al., 2021). The authors of the cited work, based on the research conducted, contrary to existing hypotheses, believe that the domestication of sheep of haplogroup A occurred on the Indian subcontinent.

Phylogenetic analysis of the D-loop mtDNA sequences of 963 individuals from 16 indigenous breeds distributed across seven geographic regions of China showed that three haplogroups, A, B, and C, were identified in all breeds, except for the mountainous region of Southwest China, where only haplogroups A and B were present (Zhao E. et al., 2013).

Seven local Indonesian sheep breeds on the island of Java are closely related to each other and grouped into two haplogroups A and B. At the same time, most of them belong to haplogroup B, except for the Garut and Priangan sheep breeds (Ibrahim et al., 2023). Sheep of the main breed of Vietnam, Phan Rang, belong to haplogroups A (28 %) and B (72 %), which confirms the hypothesis of their dual origin with the influence of both Asian and European lines (Luong et al., 2024). Phylogenetic analysis of six breeds of sheep in Kazakhstan showed that 44 % of the animals belonged to haplogroup A, 39 % to haplogroup B, and 17 % to haplogroup C, which is consistent with the distribution of haplogroups in ancient domestic sheep. Currently, line A predominates in Kazakh sheep breeds. This indicates that most Kazakh sheep breeds are genetically closer to the Asian genotype than to the European one (Tarlykov et al., 2021).

Sheep in Africa belong to different mitochondrial lineages, which gives them an advantage in genetic diversity of breeds and populations, and has practical significance for use in breeding programs to increase productivity and adaptive traits. Meta-analysis of 399 breeds allowed the identification of three main haplogroups (A, B, and C), with haplogroup B being the dominant one (Wanjala et al., 2021; Salim et al., 2023).

Currently, 17 fine-wool breeds (57.9 % of the total sheep population) are raised in Russia, as well as 17 semi-fine-wool breeds (4.1 %), 2 semi-coarse-wool breeds (1.1 %), 16 coarsewool breeds (30.3 %), and unidentified breeds (6.6 %) (Brigida et al., 2025). Semi-fine-wool sheep breeds mainly have a meat-wool production direction, while semi-coarse-wool and coarse-wool breeds have a meat-fat direction. The abundance of sheep breeds in the country is associated with the diversity of natural-climatic and geographical zones.

Genetic analysis of mtDNA of ancient sheep from Russia indicates that during the Early Bronze Age, at least two lineages (A and B) existed in the south of Western Siberia (Dymova et al., 2019). Sequencing of the mitochondrial DNA D-loop from 17 sheep bone remains (aged ~4,000–1,000 years), found in archaeological complexes in southern Altai (Russia), showed the presence of mitochondrial lineages A, B, C, D, and E. The relatively high diversity of sheep haplotypes, including the presence of two basal haplotypes, indicates that the Altai region could have been a route for the migration of domesticated sheep. Determining the affiliation of ancient samples to phylogenetic lines showed that 70 % belong to line B, 25 % to A, and 2.5 % each to C and D (Dymova et al., 2017).Phylogenetic analysis of 25 Russian local sheep breeds revealed four haplogroups, including A, B, C, and D, which is explained by the wide range of the animals studied; the most common were haplogroups B and A, characteristic of sheep of European and Asian origin, with the B lineage predominating in the western part of Russia (Koshkina et al., 2021).

Analysis of the median-joining network and the Bayesian tree of the Russian breeds showed that the most common haplogroup is B (64.8 %), haplogroup A accounted for 28.9 %, haplogroup C, for 5.5 %, and only 0.8 % was assigned to haplogroup D. It is noted that haplogroups A and B were present in all sheep breeds, haplogroup C was represented by haplotypes of sheep breeds from the North Caucasus and Far Eastern Federal Districts, while haplogroup D was detected in one animal from the Southern Federal District (Koshkina et al., 2023). M. Tapio et al. (2006) discovered haplogroup D in one animal of the Karachay breed in the North Caucasus, indicating the presence of this mitochondrial line in the territory of Russia.

The study of the nucleotide sequence of the D-loop of mtDNA in fine-wool sheep breeds (Salsk, Stavropol, and Soviet Merino) showed the division of the studied sheep populations into two clusters: the first cluster is represented by haplogroup B, characteristic of European domestic sheep, as well as sheep from New Zealand and Australia; the second cluster is haplogroup A (about 30 %), dominant in Asian sheep. The obtained research results made it possible to establish the similarities and differences of the Russian Merino sheep breeds with the European and Australian Merino selection (Shirokova et al., 2018). In the determination of the Romanov sheep (Bakoev et al., 2018), the Kuibyshev breed (Koshkina et al., 2022a), it was established that they belong to haplogroup B.

Analysis of the mitochondrial genomes of three breeds with different production directions (Grozny – fine-wool, Gorno- Altai – semi-fine-wool, and Buubey – coarse-wool) from various ecological and feed regions of the country showed that all individuals of the three breeds belong to the commonly accepted haplogroups A (n = 46.7 %), B (n = 46.7 %), and C (n = 6.6 %), characteristic of sheep of European and Asian origin (Koshkina et al., 2022b).

## Conclusion

Thus, based on the analysis of scientific and informational sources in the subject area, it has been established that with domestication, aspects such as the specific place, time, and further development after domestication remain unclear to this day.

Mitochondrial DNA is an excellent tool of evolutionary biology. It is inherited from generation to generation almost without changes, since it does not participate in recombination, thus the time of occurrence of each mutation can be estimated based on the average mutation rate. This ensures that female haplogroups allow us to determine events that occurred in the distant past (processes of domestication and migration). Seven haplogroups have been identified in domesticated sheep, two of which (F and G) have disappeared, while the other five (A, B, C, D, E) are present in modern breeds.

The variability of mtDNA to some extent correlates with the distance from the domestication center. The greatest genomic diversity is found closer to the place of origin, as has been established in humans (Li J.Z. et al., 2008). This review confirms the existence of a single center of domestication in the Middle East, from which sheep spread to Europe through the Mediterranean Sea and the Danube Valley, as evidenced by studies of small ruminant lentiviruses (Molaee et al., 2020). It is possible that there was another way of spreading sheep into Europe, which passed through the Caucasus, Russia, and Northern Europe (Tapio et al., 2006). First, line B probably reached Finland, then, in the early Middle Ages, it was followed by line A (Niemi et al., 2013). The origin of lines C and D in Central Europe remains unclear, but there is a suggestion that they could have arrived there with prehistoric humans or much later, for example, during the Ottoman expansion (Gáspárdy et al., 2021). The Mongolian Plateau acted as a migration center, from which the lines spread from the domestication center into Asia (Ganbold et al., 2019). In India, a high nucleotide diversity of lines A and B has been established, in Northern China, line C has been found (Lv et al., 2015).

During the process of migration, domesticated sheep adapted to various ecological and feeding conditions of their habitats on all continents of the world, and as a result, modern domestic sheep have developed a number of morphological, physiological, and behavioral traits, which have led to the current phenotypic differences between wild and domestic species.The results of ancient DNA studies have shown that Anatolian Neolithic sheep have more similarity with modern European breeds, while Kyrgyz sheep of the Neolithic and Bronze Age demonstrate more similarity with modern Asian breeds.

Currently, domestic sheep are divided into two main geographical groups, Europe and Asia: European sheep mainly carry haplotype B, while Asian sheep predominantly carry haplotype A. The diversity of the haplogroups represented in both domesticated and modern breeds of sheep indirectly indicates their migration across the territory of Eurasia, including the Russian Federation, in two directions – to Europe (westward) and to Asia (eastward).

## Conflict of interest

The authors declare no conflict of interest.
